# Giant Pseudoaneurysm Associated with Arteriovenous Fistula of the Brachial and Femoral Arteries following Gunshot Wounds: Report of Two Cases

**DOI:** 10.1155/2015/454713

**Published:** 2015-02-03

**Authors:** Handy Eone Daniel, Ankouane Firmin, Pondy O. Angele, Minka Ngom Esthelle, Bombah Freddy, Ngo Nonga Bernadette

**Affiliations:** ^1^Department of Surgery, Faculty of Medicine and Biomedical Sciences, University of Yaoundé I, Yaoundé, Cameroon; ^2^Department of Medicine, Faculty of Medicine and Biomedical Sciences, University of Yaoundé I, Yaoundé, Cameroon; ^3^Department of Pediatrics, Faculty of Medicine and Biomedical Sciences, University of Yaoundé I, Yaoundé, Cameroon

## Abstract

Posttraumatic pseudoaneurysm associated with arteriovenous fistula of the upper or lower limb is exceptional. We are reporting herein the history of two cases in civil life that have been followed and repaired in our service. Both patients were shot more than a year before being referred to our tertiary hospital for an enlarging mass which was a pseudoaneurysm associated with an arteriovenous fistula. The aneurysm was repaired and the fistula closed. Due to the absence of well-trained professionals, vascular injuries and their complications are usually discovered late in Cameroon while these pseudoaneurysms can reach very dramatic sizes. This presentation intends to raise the attention on a careful clinical exam and search of vascular lesion in the case of penetrating wound of the limb associated with profuse bleeding.

## 1. Introduction

Isolated posttraumatic pseudoaneurysms of the peripheral vessels have been reported in the literature and are more common during war time [1]. Pseudoaneurysms associated with arteriovenous fistula (AVF) are uncommon following penetrating wound trauma of the limbs and have rarely been reported. They have been found more on the upper extremities than the lower extremities [2]. In Africa, few studies on vascular trauma and its complications have been reported [3]. Due to the increasing violence in the fast growing cities in Cameroon, vascular traumas are becoming more common [4]. Yet many of these lesions go unrecognized and are diagnosed at the stages of late complications like pseudoaneurysm and arteriovenous fistula. The combination of both complications can result in a severe treat to the extremity involved [2, 5]. We are reporting two cases of brachial and femoral arteries' pseudoaneurysm associated with arteriovenous fistula to the corresponding venous system, occurring more than a year after gunshot wounds. The following presentation emphasizes the need of careful examination of a patient with penetrating injury near major vessels of the limbs and the development of vascular clinical exam.

**Figure 1:**
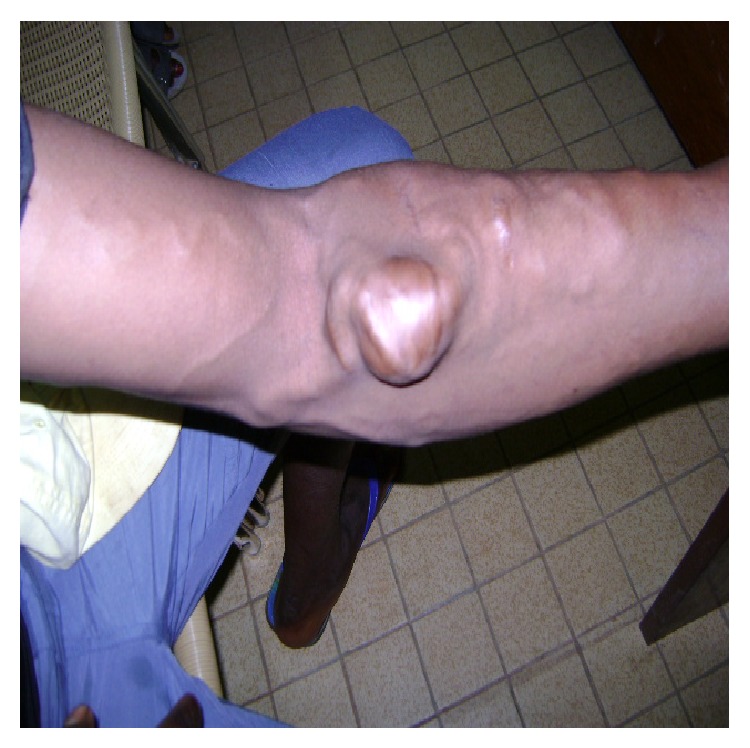
Posttraumatic AVF between the brachial artery and the vein comitans with a large pseudoaneurysm and massively distended superficial veins; the distension is from the venous comitans.

**Figure 2:**
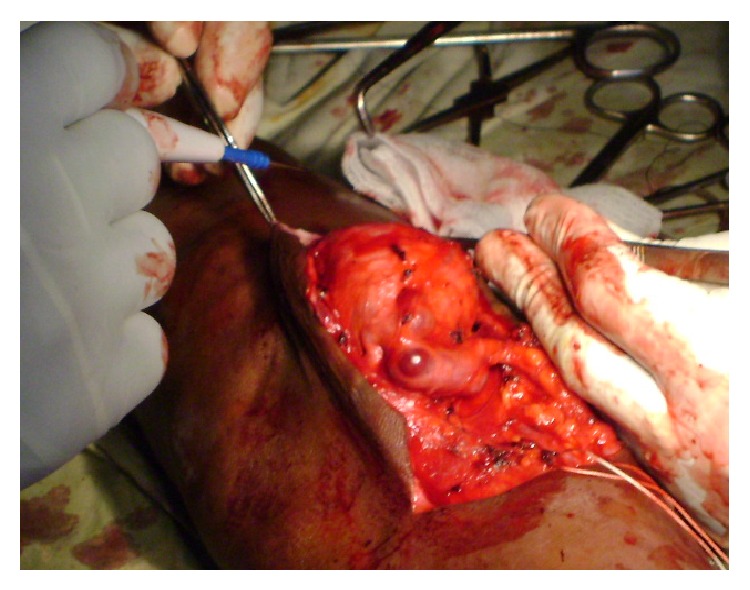
Dissection of the pseudoaneurysm. The elastic band is around the brachial artery. The vessels are massively distended. The fistula was between the venous comitans and the brachial artery. So the pseudoaneurysm was just under the skin but having a large communication with the brachial artery.

**Figure 3:**
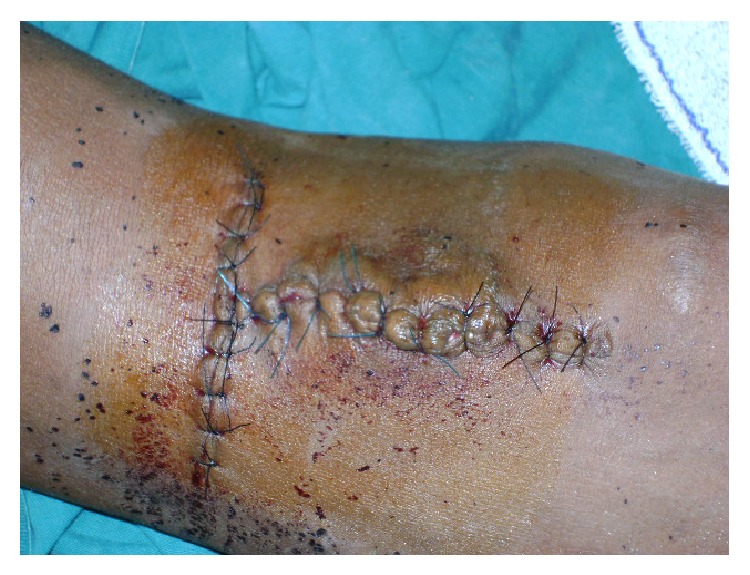
Immediate postoperative appearance of the elbow. The distended veins have disappeared.

*Patient 1.* This 22-year-old male farmer from a village (150 Km from Yaoundé) was referred to us for a pulsating mass of the left elbow, swollen upper limb, and massively distended veins associated with mild pain. He reported that he was shot with a shotgun 2 years earlier while hunting. He was taken to the hospital just after the injury because he was bleeding profusely. Scattered small wounds were found, a dressing was done, and he was transfused 2 units of blood. There was no other injury elsewhere. He was discharged home with no referral or follow-up appointment. He decided to see a doctor 2 years later because of an enlarging mass of the left elbow. On the physical exam, there was a 6 cm × 8 cm pulsating mass at the left elbow and massively distended superficial veins; the radial pulse was present but weak (Figure 1). The left upper limb was mildly swollen and warmer than the right one. There was a strong pulsating and audible thrill at the level of the mass and also a machinery sound audible at the level of the superficial veins. The diagnosis of a posttraumatic pseudoaneurysm of the brachial artery associated with an AVF was made. The young man was very poor and had no money to do any additional work-up test. Because he was a farmer and needed to use his limb, we decided to help him and performed the surgery under local anesthesia. We first controlled the brachial artery just above the elbow and the distal control was obtained just after the pseudoaneurysm (Figure 2); we explored and dissected around the pseudoaneurysm without disrupting it. We found a fistula between the vein comitans (anastomosing the cephalic vein to the brachial vein) and the brachial artery after the elbow. Ligation of the superficial veins was realized, the fistula was divided, and the artery and the veins were repaired using the prolene 6/0. The blood loss was minimal and no transfusion was necessary. The patient was discharged home the same day and was followed in our clinics.  Figure 3 shows the postoperative clinical aspect of the limb.

**Figure 4:**
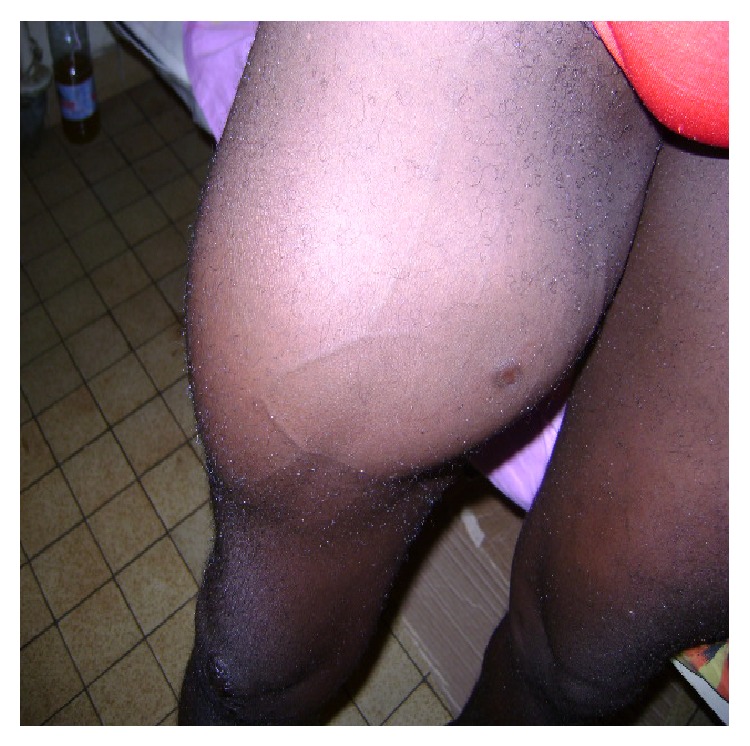
Large superficial femoral pseudoaneurysm with AVF fistula with palpable trill and audible machinery murmur.

*Patient 2.* This young man was a 27-year-old student when he was assaulted and shot on the right thigh. He was taken to a major hospital in Douala. The wounds were found to be small, so no suturing was done and no other exam was requested. The wounds were dressed daily. We have no description of the physical exam at that time. One year later after the incident, he was referred to us complaining of an enlarging mass in the middle of the right thigh associated with intermittent claudication (Figure 4). These symptoms which started after the accident have been increasing gradually and walking for him was more and more difficult. When we saw him for consultation, he was having rest pain, difficulties in walking, and impotence of the right lower limb and he was using crunches to walk. This pain was not responding to codeine based pain medication and was found to be 9/10 in the analogic scale; the pain increased by walking and was located more at the level of the calf. He also complained of paresthesia and mild paralysis. The walking distance has tremendously decreased the last month before consultation and he was feeling pain while being at rest.

**Figure 5:**
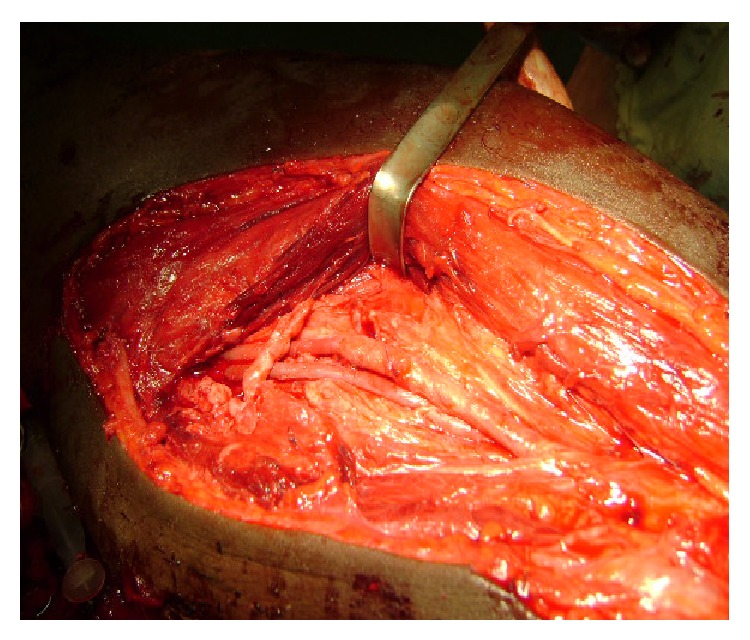
Superficial femoral artery and vein after repair using saphenous vein replacement. We have used the inverted larger portion of the vein to repair the artery and the smaller portion to repair the vein.

On the physical exam, he presented a large mass located on the right thigh measuring 12 × 9 cm; it was warm, pulsating with an audible and palpable thrill located in the internal surface of the right thigh in the upper third. Besides the thrill, a large machinery murmur was also audible at the level of the Scarpa triangle. We noticed also two small old scars as shown in Figure 4. The foot was cold, and no distal pulse was palpated (popliteal, posterior tibial, or pedal pulse); he had a decreased sensation of the foot and was unable to dorsiflex it. An angioscan showed a large pseudoaneurysm with communication between the femoral vessels. The diagnosis of post traumatic pseudoaneurysm of the superficial femoral artery associated with an AVF between this artery and the corresponding vein complicated by ischemia of the right leg was made. The patient was taken to the operating room on an urgent basis because of impending limb loss due to ischemia. The surgery was done under locoregional anesthesia. We first obtained vascular control at the level of the Scarpa triangle; after a difficult dissection, we were able to find the fistula between the superficial femoral artery and vein. The injured portions of both vessels were resected; the artery and the vein were repaired using saphenous vein grafting (Figure 5). Postoperatively, the patient did not complain of any pain at rest and the foot became warm; there was also a net improvement of the paresthesia, and he was able to dorsiflex the ankle. But no distal pulse was palpated on the right ankle and foot (from the posterior tibial or pedal arteries).

## 2. Discussion

In Cameroon, some trauma patients are being taken care of by nursing staff (first patient) or general practitioner (second case) who are not trained in vascular injury's clinical presentation. Therefore, with penetrating injuries, patients are usually seen at the time of the trauma; most of them are even transfused blood because of profuse bleeding but the diagnosis is not suspected and the appropriate exams or referrals are not requested. The two cases reported here are illustrative of these facts; the diagnosis was delayed although they have been seen in hospitals at the time of the lesions. Delayed diagnosis is also secondary to the poverty of these patients, very few being able to pay for the exams or the treatment in a tertiary hospital where this diagnosis can be made.

As reported by others, an injury to a peripheral artery should be suspected when one of these clinical signs is present: a penetrating injury next to a major artery, profuse bleeding, expanding hematoma, signs of peripheral ischemia, shock with a need for transfusion, and obvious signs of vascular lesions. All of these signs were found in both patients and it is unlikely that the diagnosis would have been missed if they were seen in a tertiary center as in our hospital [1, 2, 5]. Late complications of peripheral artery's injuries are pseudoaneurysms and AVF. Each complication, though rare, is not uncommon independently [1, 3], but the association of a pseudoaneurysm and AVF has not been reported in Cameroon and is quite rare in the literature. These lesions are easily recognized in a patient with a pulsating mass, associated with distended superficial veins; the extremity is warm, a thrill is palpable, and a machinery murmur is audible. The superficial veins are distended; long standing cases can lead to massive enlargement of both the arteries and the veins involved [6]. In both cases presented here, the extremity was warm and the arteries and veins were massively distended. Although the diagnosis is obvious based on the clinical exam, a duplex ultrasound may be useful. Other exams are MRI angiogram or angioscan. An arteriography may be useful, but it is not available in Cameroon and has been replaced in many cases by MRI angiogram in western countries, because it carries more complications [1, 2, 5]. Complications like rupture and ischemia are life-threatening. To avoid limb loss, rupture, infection, and delayed cardiac failure, the surgery should be realized without delay.

Late complications of vascular injuries due to a shotgun are pseudoaneurysm, arteriovenous fistula, and a combination of both. The aneurysm can reach dramatic proportion with the threat to the life of the patient (Figures 1 and 3). Long standing lesions can also lead to massively distended veins and arteries as in our first case. A high suspicion at the time of the injury is advisable, and a vascular consultation should be sought in any patient with a penetrating injury in the extremity presenting with profuse bleeding and shock even if he does not have signs of ischemia.
